# Dietary supplementation with seed oil from transgenic *Camelina sativa* induces similar increments in plasma and erythrocyte DHA and EPA to fish oil in healthy humans

**DOI:** 10.1017/S0007114520002044

**Published:** 2020-11-14

**Authors:** Annette L. West, Elizabeth A. Miles, Karen A. Lillycrop, Lihua Han, Johnathan A. Napier, Philip C. Calder, Graham C. Burdge

**Affiliations:** 1 School of Human Development and Health, Faculty of Medicine, University of Southampton, Southampton SO16 6YD, UK; 2 Centre for Biological Sciences, Faculty of Natural and Environmental Sciences, University of Southampton, Southampton SO17 1BJ, UK; 3 Department of Plant Sciences, Rothamsted Research, Harpenden AL5 2JQ, UK; 4 NIHR Southampton Biomedical Research Centre, University Hospital Southampton NHS Foundation Trust and University of Southampton, Southampton SO16 6YD, UK

**Keywords:** Transgenic plants, *Camelina sativa*, DHA, EPA, Dietary supplementation

## Abstract

EPA and DHA are required for normal cell function and can also induce health benefits. Oily fish are the main source of EPA and DHA for human consumption. However, food choices and concerns about the sustainability of marine fish stocks limit the effectiveness of dietary recommendations for EPA + DHA intakes. Seed oils from transgenic plants that contain EPA + DHA are a potential alternative source of EPA and DHA. The present study investigated whether dietary supplementation with transgenic *Camelina sativa* seed oil (CSO) that contained EPA and DHA was as effective as fish oil (FO) in increasing EPA and DHA concentrations when consumed as a dietary supplement in a blinded crossover study. Healthy men and women (*n* 31; age 53 (range 20–74) years) were randomised to consume 450 mg/d EPA + DHA provided either as either CSO or FO for 8 weeks, followed by 6 weeks washout and then switched to consuming the other test oil. Fasting venous blood samples were collected at the start and end of each supplementation period. Consuming the test oils significantly (*P* < 0·05) increased EPA and DHA concentrations in plasma TAG, phosphatidylcholine and cholesteryl esters. There were no significant differences between test oils in the increments of EPA and DHA. There was no significant difference between test oils in the increase in the proportion of erythrocyte EPA + DHA (CSO, 12 %; *P* < 0·0001 and FO, 8 %; *P* = 0·02). Together, these findings show that consuming CSO is as effective as FO for increasing EPA and DHA concentrations in humans.

*n*-3 PUFA, namely EPA (20 : 5*n*-3) and DHA (22 : 6*n*-3), are present primarily in oily fish and are important for development and tissue function. DHA is important for the development and function of the central nervous system, including the retina^(1,2)^. EPA is a substrate for the synthesis of a range immunomodulatory second messengers^(3)^. Increased EPA + DHA intake can convey health benefits such as reduced inflammation, and lower postprandial and fasting blood TAG concentration^(4)^. Moreover, the sum of the proportions of EPA + DHA in erythrocytes, the omega-3 index, is a biomarker of CVD risk^(5)^.

Although humans are able to synthesise some EPA and DHA from the essential fatty acid *α*-linolenic acid (ALA, 18 : 3*n*-3) found in vegetable oils, the activity of this pathway is low and its contribution to meeting demands is not known^(6)^. Consequently, humans rely primarily on consumption of pre-formed EPA and DHA in their diet. Some governments and advisory organisations have published guidelines and recommendations for EPA and DHA intake. For example, the UK government recommends that all adults should consume 450 mg EPA + DHA/d to maintain health^(7)^. However, the effectiveness of such recommendations is constrained by dietary choices of the population. For example, only about 27 % of UK fish consumers eat oily fish and overall UK adults consume <200 mg EPA + DHA/d, while children consume approximately one-tenth of the adult intake^(8,9)^. Such avoidance of oily fish reflects, at least in part, the cost of oily fish, perceived unpalatability and concerns about contamination with environmental pollutants^(8,9)^. Vegetarians and vegans who exclude fish and meat, or fish, meat and dairy products from their diet have approximately 50 % lower EPA and DHA levels in blood compared with omnivores^(10)^. In addition to the limited compliance to recommendations for EPA + DHA by the UK population, consumption of oily fish and, in particular, the production of fish oils (FO) represent a substantial demand on marine ecosystems. Even at present levels, the total marine production of EPA and DHA from oily fish has been estimated to be approximately 1·1 million metric tonnes less than that required to meet the needs to the global population for EPA and DHA^(11)^. Hence, there is a need for alternative sustainable, affordable and scalable sources of EPA and DHA that are compatible with the full range of human dietary choices.

Alternative sources of EPA and DHA to oily fish include krill which produces a yield of EPA + DHA equivalent to 0·3 % of the global production of these fatty acids and algal oils which account for <2 % global EPA + DHA production^(11)^. Increasing production of EPA + DHA from krill has raised concerns about possible negative impact on the ecology of the South Atlantic^(11)^. The cost of increasing the scale of EPA + DHA production from algae is likely to be prohibitive, and most commercial species have a high DHA content (20–48 % total fatty acids), but with an EPA content below 1 %^(12)^. Vegetable oils that the contain ALA are unlikely to be an effective means of meeting the demands for EPA and DHA in humans because of low capacity for ALA interconversion^(6)^.

Genetic modification of oil seed plants is potentially a sustainable means of producing EPA and DHA for human consumption that is consistent with dietary choices that exclude animal-derived foods and does not incur concerns about environmental contaminants or palatability. Strains of transgenic *Camelina sativa* and rapeseed have been developed that produce seed oils which contain EPA and DHA^(12)^, of which one strain of transgenic *C. sativa* produced a seed oil that approximated the EPA and DHA content of marine FO^(13)^. To date, the acute bioavailability of EPA and DHA in humans when consumed as the seed oil from a transgenic plant has only been tested for this one transgenic *C. sativa* strain. The findings showed that there was no significant differences in men and women aged 18–30 years or 50–65 years in the postprandial incorporation of EPA and DHA into blood lipids between FO and transgenic *C. sativa* seed oil (CSO) that both contained approximately 12 % EPA and 9·5 % DHA^(14)^. Thus, acute consumption of this CSO appears to be as effective as FO as a source of EPA and DHA in humans. However, the effectiveness of CSO in raising blood EPA and DHA concentrations when consumed as a dietary supplement has not been tested. To address this, we compared the incorporation of EPA and DHA into blood lipids when consumed for 8 weeks as either a commercial FO or CSO in healthy adults in a randomised crossover study.

## Materials and methods

### Preparation of *Camelina sativa* oil

Transgenic *C. sativa* plants producing a seed oil containing EPA and DHA were generated as described^(14)^. Homozygous T3 generation transgenic *C. sativa* plants were grown in a controlled environment containment glasshouse under long-day conditions (16 h light–8 h dark), 50–60 % relative humidity, with temperature 23°C d/18°C night, and with a light intensity of 400 μmol/m^2^ per s. Seeds were harvested and threshed, and the oil was then extracted^(14)^. Further processing by refining, bleaching and deodorising was carried out by POS Bio-Sciences.

### Human dietary supplementation study

The study was reviewed and approved by the South Central – Hampshire B Research Ethics Committee (REC reference 15/SC/0627). The trial is registered at ClinicalTrials.gov (identifier: NCT03477045). All participants gave written informed consent.

The participants were healthy men and women whose characteristics are summarised in Table 1. The inclusion criteria for the study were to be 18–75 years of age, with BMI 18·5–30·0 kg/m^2^, to be within normal clinical ranges for blood pressure, to have total cholesterol and non-fasting glucose concentrations within accepted ranges, to not habitually consume FO or other dietary oil supplements, or not eating more than one oily fish meal per week, to be willing and able to adhere to the study protocol, and able to provide written informed consent. Volunteers were excluded if they had a BMI >30 kg/m^2^, clinician diagnosed chronic illness or food allergy, were regular user of anti-inflammatory medication or had been prescribed medication to control blood lipid concentrations or fat absorption, or to control blood pressure or chronic gastrointestinal disease. Volunteers were also excluded who were pregnant or planning to become pregnant during the study period or were participating in another clinical trial. Because we have shown that there were no significant differences between sexes or between ages in acute incorporation of EPA and DHA into blood lipids^(14)^, the participants were studied as a single group of mixed ages and sexes.

**Table 1.** Characteristics of participants at enrolment* (Mean values with their standard errors; medians and ranges)

	Mean		sem
Proportion of females (%)		56	
Age (years)			
Median		53	
Range		20–74	
Height (m)	1·72		0·02
Weight (kg)	72·4		2·5
BMI (kg/m^2^)	24·5		0·5
Systolic blood pressure (mmHg)	117·7		23
Diastolic blood pressure (mmHg)	69·1		1·5
Fasting plasma glucose (mmol/l)	5·1		0·1
Total plasma cholesterol (mmol/l)	4·9		0·2
Total plasma TAG (mmol/l)			
Median		0·8	
Range		0·2–2·9	

* Values for each characteristic are shown for thirty-one participants.

The trial had a blinded crossover design. After health screening by questionnaire and by measurement of anthropometric and biochemical markers, participants were randomised to consume 450 mg EPA + DHA/d provided either by the CSO or a commercial blended FO (Simply Timeless®, Omega-3 FO plus cod liver oil; Seven Seas) in random order for a period of 8 weeks followed by a washout interval of 6 weeks. Randomisation was carried out using a random number generator (www.random.org). Participants were instructed not to consume more than one fish meal per week, and if fish were consumed, then it should not be oily. Compliance was assessed verbally on each study visit, and there was no evidence of non-compliance. Participants then consumed the other test oil for a further 8 weeks. The fatty acid compositions of the oils are detailed in Table 2. Blinding was achieved by dispensing the test oils into identical containers that were filled and labelled by a researcher who was not a member of the study team. However, blinding was incomplete because the FO retained some residual odour and taste. Participants were instructed to dispense the appropriate volume of test oil (CSO, 2·4 ml; FO, 1·6 ml) using an oral dosing syringe and to consume this once per d in the morning just before breakfast so that the oil would mix with ingested food and also induce an insulinogenic response required to promote hydrolysis of dietary lipids in blood. Compliance was assessed by weighing the individual bottles containing the test oils before and at the end of each supplementation period and then comparing the difference to the change in weight expected if the correct amount of oil was withdrawn each day.

**Table 2.** Fatty acid compositions of the test oils

	Proportion of total fatty acids (% total fatty acids)	
Fatty acid	CSO	FO
14 : 0	0·1	6·5
16 : 0	6·6	15·0
16 : 1*n*-7	0·2	9·0
18 : 0	5·5	2·9
18 : 1*n*-9	5·9	14·5
18 : 1*n*-7	1·6	4·1
18 : 2*n*-6	19·9	1·6
18 : 3*n*-6	3·1	0·2
18 : 3*n*-3	13·6	1·1
20 : 0	2·9	0·2
20 : 1*n*-9	6·1	6·6
20 : 2*n*-6	0·9	0·3
20 : 3*n*-6	0·9	0·2
20 : 4*n*-6	2·9	1·0
20 : 4*n*-3	2·8	6·2
EPA	10·8	16·0
24 : 0	1·0	0·1
22 : 5*n*-3	6·6	1·9
DHA	8·6	12·9
EPA + DHA	19·4	28·9
Total SFA	16·1	24·7
Total MUFA	13·8	34·1
Total *n*-6 PUFA	27·6	3·2
Total *n*-3 PUFA	42·4	38·0

CSO, *Camelina sativa* seed oil; FO, fish oil.

Venous blood samples (40 ml) were collected using lithium heparin anticoagulant after participants fasted for 12 h at the start and end of each supplementation period. The blood samples were separated into plasma and cell fractions^(15)^. Plasma and erythrocytes were stored at −80°C before analysis of fatty acid composition.

### Analysis of the fatty acid composition of blood and erythrocyte lipids

We have shown that the structures of EPA and DHA, containing TAG, differ between FO and CSO which may, in turn, modify the incorporation of these fatty acids into plasma lipid classes^(16)^. We, therefore, undertook a comprehensive analysis of the incorpration of EPA and DHA into the main plasma lipid classes. The fatty acid composition of plasma TAG, phosphatidylcholine (PC), cholesteryl esters (CE) and NEFA was determined by GC as described^(15,17)^. Briefly, the internal standards dipentadecanoyl PC (100 μg), triheptadecanoin (100 μg), heneicosanoic acid (50 μg) and cholesteryl heptadecanoate (100 μg) were added to plasma (0·8 ml) and total lipids and then extracted with chloroform–methanol (2:1, v/v)^(17,18)^. Individual lipid classes were isolated by solid phase extraction as described previously^(17)^ on a 100 mg aminopropylsilica column (BondElut; Agilent Technologies)^(19)^. Fatty acid methyl esters (FAME) were prepared by reaction of isolated lipids with methanol containing 2 % (v/v) sulphuric acid at 50°C for 2 h^(17)^. FAME were resolved on a BPX-70 fused silica capillary column (30 m × 0·25 mm × 25 μm) using an Agilent 6890 gas chromatograph equipped with flame ionisation detection^(15)^. Fatty acids were identified by their retention times relative to standards (37 FAMES, Sigma-Aldrich). The concentrations of individual FAME were determined by comparison of the peak area with that of the internal standard with adjustment for the volume of plasma that was extracted.

Erythrocytes were extracted with chloroform:methanol 2:1 as described^(20)^. The fatty acid composition of total erythrocyte lipids was determined by GC using the same method as used for plasma lipids. The fatty acid composition of the test oils was determined as described^(14)^.

### Measurement of the size and concentration of lipoproteins

Determination of the concentration and diameter of chylomicrons (CM), VLDL, LDL, intermediate-density lipoprotein and HDL particles was carried out using NMR spectroscopy by LipoScience Incorporated as described previously^(14)^. Because the size of VLDL and CM particles can overlap, the size distributions and concentrations of these particles are reported as a combined VLDL + CM fraction which in fasting samples were likely to contain predominately VLDL and a smaller proportion of CM remnants.

### Measurement of plasma total TAG and glucose concentrations

Plasma TAG, NEFA and glucose concentrations were measured using a Konelab 20 autoanalyser (Labmedics Ltd) as described^(21)^. Reagents were from Microgenics GmbH and Alpha Laboratories.

### Statistical methods

There are currently no other dietary supplementation studies involving the CSO on which to base a calculation of sample size. Consequently, we used the findings of our previous study^(22)^ to estimate the statistical power of this exploratory study. Thirty-one participants were estimated, using online calculators at www.dssresearch.com, to provide 85 % power for detecting a 4 % difference in the primary end point, DHA concentration in plasma PC between test oils, at *α* = 5 % in paired two-tailed analysis. Data were analysed using the SPSS statistical analysis program (IBM Corp. (released 2017) IBM SPSS Statistics for Windows, version 25.0). Any effects of the order in which the oils were consumed were tested by Student’s paired *t* test of the absolute changes in concentration during each period of supplementation^(23)^. If no order effects were found, data were pooled for each type of oil irrespective of the order in which the oil was consumed and the effects of the test oils on EPA and DHA concentrations were analysed using Student’s paired *t* test^(23)^. Data which were not normally distributed were analysed using the Mann–Whitney *U* test. Adjustment for multiple testing was carried out for data sets that showed a statistically significant unadjusted *P* value. Associations between data sets were tested by calculating Pearson’s correlation coefficient.

## Results

### Participant recruitment, tolerance of the trial and compliance

**Fig. 1.**
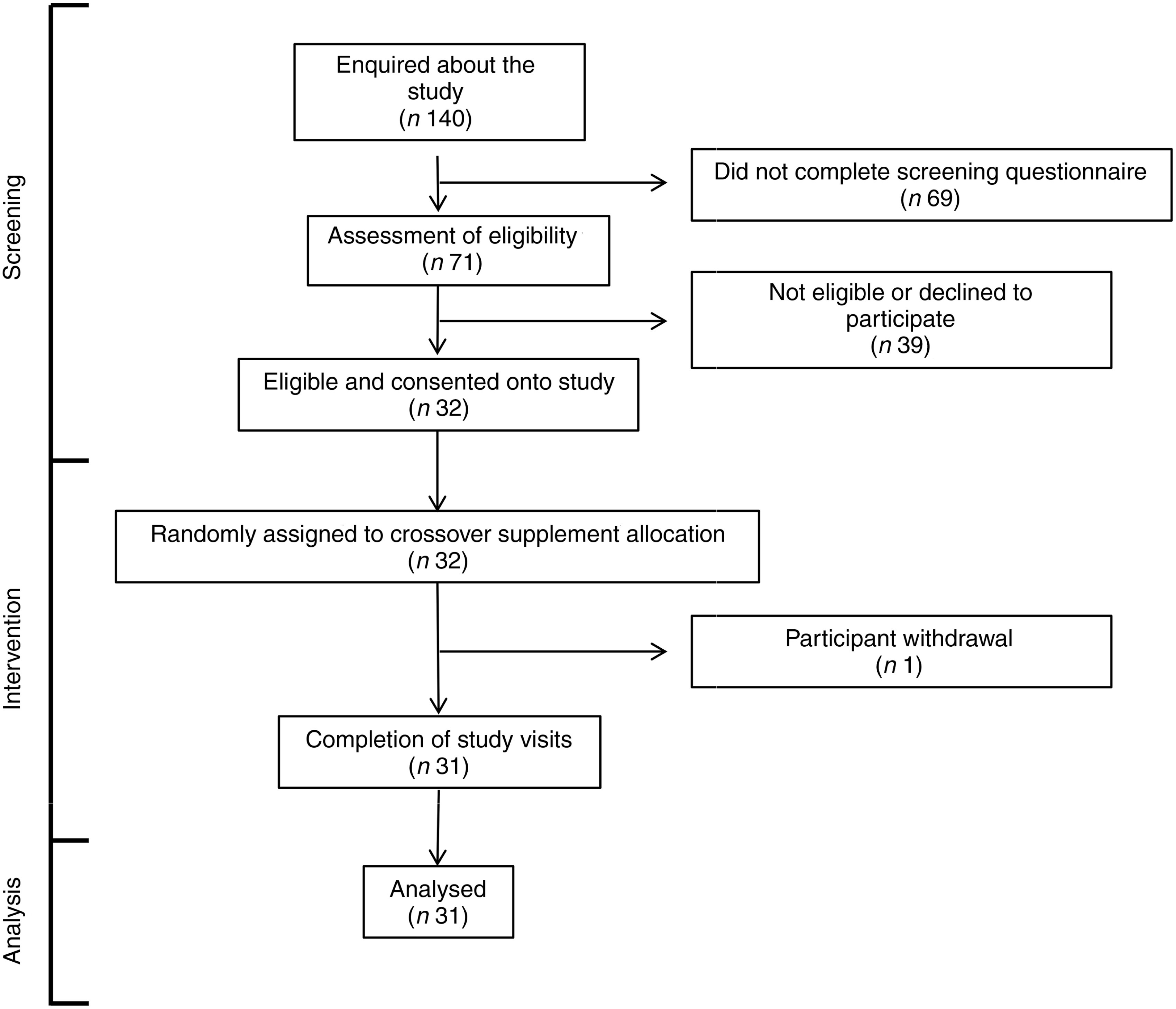
CONSORT diagram of the destinations of volunteers.

A total of 140 individuals enquired about the study, of which sixty-nine did not complete the screening questionnaire for undisclosed reasons (Fig. 1). Seventy-one of the remaining individuals were assessed for eligibility against study inclusion criteria. Thirty-nine of these were found to be ineligible or decided not to participate. The remaining thirty-two volunteers gave written informed consent to take part in the study and were randomised to one of the two test oils. One participant withdrew after commencing the supplementation due to perceived unpalatability of the FO supplement. Thirty-one participants completed both supplementation periods. The median compliance to the supplementation protocol was FO 97 (range 71–103) % and CSO 99 (range 82–113) %. There was no statistically significant difference in compliance (*P* = 0·2) between test oils by the Wilcoxon matched-pairs signed-rank test. Some participants who exceeded 100 % compliance may have done so by consuming more than the intended amounts of oils. The volunteer with the highest compliance value consumed 113 % of the intended amount which is equivalent to an extra 38 mg/d EPA and 30 mg/d DHA over the 8 weeks supplementation period. There was no significant effect of the order in which test oils were consumed on the incorporation of EPA or DHA into plasma lipids (all *P* > 0·05) or on any of the other outcomes that were measured. Therefore, data from each arm of the crossover study were pooled according to the test oil that was consumed. There were no significant differences between male or female participants in the change from baseline EPA or DHA concentrations after consuming the test oils (all *P* > 0·05).

One participant withdrew from this study (Fig. 1). All other participants tolerated supplements well. Three adverse events were reported during intervention, including one case of ‘fishy burps’ when consuming FO, one case of exacerbation of pre-existing dermatitis and one case of stomach irritation that did not appear to be due to either of the test oils.

### The effect of dietary supplementation with fish oil or *Camelina sativa* seed oil on EPA and DHA concentrations in plasma lipids

Consuming either FO or CSO for 8 weeks significantly increased plasma PC EPA concentration by 49 and 79 %, respectively, compared with baseline (Table 3). Consuming FO or CSO for 8 weeks increased EPA concentration in plasma TAG by 50 and 98 %, respectively (Table 3). Consuming CSO for 8 weeks significantly increased EPA concentration (29 %) in plasma NEFA (Table 3), while the increase following consumption of FO (49 %) failed to reach statistical significance. Consuming FO or CSO for 8 weeks significantly increased EPA concentration by 52 and 49 %, respectively, in plasma CE. There was no statistically significant difference between test oils in the increment in EPA concentration in either plasma NEFA or CE.

**Table 3.** Effect of dietary supplementation with fish oil (FO) or *Camelina sativa* seed oil (CSO) on EPA and DHA concentrations in plasma lipids* (Mean values with their standard errors; *n* 31)

	Concentration (μmol/l)							
	Start		End		Difference		Change from baseline	Δ FO *v*. Δ CSO
	Mean	sem	Mean	sem	Mean	sem	*P*	*P*
EPA								
PC								
FO	29·4	2·9	43·7	4·8	14·3	4·0	0·001	0·3
CSO	28·5	3·5	49·6	6·8	21·1	5·2	<0·0001	
TAG								
FO	5·0	0·7	7·5	0·9	2·5	0·8	0·003	0·3
CSO	4·9	0·6	9·7	2·0	4·8	1·8	0·01	
NEFA								
FO	0·7	0·1	0·9	0·1	0·2	0·1	0·07	0·5
CSO	0·7	0·1	1·0	0·1	0·3	0·1	0·006	
CE								
FO	27·8	3·5	42·3	5·9	14·4	4·3	0·002	0·3
CSO	27·2	3·8	48·6	7·0	21·4	4·9	0·02	
DHA								
PC								
FO	80·2	5·6	96·2	8·9	15·9	4·8	0·003	0·9
CSO	82·0	8·5	98·6	9·1	16·6	5·3	0·004	
TAG								
FO	7·3	1·0	10·3	1·1	3·0	1·0	0·005	0·4
CSO	8·2	1·3	14·1	3·8	5·9	3·4	0·09	
NEFA								
FO	2·7	0·5	3·0	0·6	0·3	0·5	0·5	0·8
CSO	2·3	0·3	2·8	0·3	0·5	0·3	0·2	
CE								
FO	11·3	1·0	14·6	1·7	3·3	1·0	0·001	0·9
CSO	12·6	1·4	15·7	2·0	3·1	1·3	0·2	

PC, phosphatidylcholine; CE, cholesteryl esters.

* All data approximated a normal distribution. The mean concentrations of EPA and DHA at the start and end of the trial and the effect of the type of oil on the mean change in EPA and DHA concentrations from baseline were analysed using Student’s paired *t* test.

Consuming either FO or CSO for 8 weeks significantly increased DHA concentration in plasma PC by 49 and 74 %, respectively, and in plasma TAG by 41 and 72 %, respectively (Table 3). Consuming FO or CSO increased DHA concentration in plasma NEFA by 11 and 22 %, respectively, although this change did not reach statistical significance. Consuming FO or CSO increased plasma CE DHA concentration by 29 and 27 %, respectively, compared with baseline, although this only reached statistical significance after consuming FO (Table 3). There were no significant differences between test oils in the magnitude of change in DHA concentration from baseline in any of the plasma lipid classes that were measured (Table 3).

### The effect of dietary supplementation with fish oil or *Camelina sativa* seed oil on plasma lipid and glucose concentrations

There were no significant effects (*P* > 0·05) of the order in which the test oils were consumed on fasting plasma glucose, or total TAG, NEFA and CE. Consuming FO induced a statistically significant decrease in plasma glucose (4 %) and TAG (20 %) concentrations (Table 4). Consuming CSO did not alter the concentrations of plasma glucose, TAG, CE or NEFA. There was no statistically significant difference between test oils in any change in plasma glucose or lipid concentrations.

**Table 4.** Effect of dietary supplementation with fish oil (FO) or *Camelina sativa* seed oil (CSO) on plasma glucose and lipid concentrations* (Mean values with their standard errors; *n* 31)

	Concentration (mmol/l)							
	Start		End		Difference		Start *v*. end	Δ FO *v*. Δ CSO
	Mean	sem	Mean	sem	Mean	sem	*P*	*P*
TAG								
FO	1·0	0·1	0·8	0·1	−0·2	0·1	0·02	0·4
CSO	0·9	0·1	0·8	0·1	−0·1	0·1	0·1	
Cholesteryl esters								
FO	2·9	0·2	3·0	0·3	0·2	0·1	0·2	0·7
CSO	3·1	0·3	3·2	0·3	0·1	0·2	0·4	
NEFA								
FO	0·3	0·1	3·0	0·1	0·1	0·1	0·3	0·4
CSO	0·1	0·1	0·1	0·1	0·1	0·1	0·9	
Glucose								
FO	4·8	0·1	4·6	0·1	−0·2	0·1	0·04	0·9
CSO	4·8	0·1	4·8	0·1	0·1	0·1	0·5	

* All data approximated a normal distribution. The mean concentrations of EPA and DHA at the start and end of the trial and the effect of the type of oil on the mean change in EPA and DHA concentrations from baseline were analysed using Student’s paired *t* test.

### The effect of dietary supplementation with fish oil or *Camelina sativa* seed oil on lipoprotein size and concentration

Dietary supplementation with either CSO or FO decreased VLDL + CM TAG concentration, although this only reached statistical significance when participants consumed FO (Table 5). There were no other significant changes in lipoprotein concentration or size between the start and end of the supplementation period. There were no significant differences between test oils in the difference in lipoprotein concentrations and size between the start and end of the intervention periods (Table 5).

**Table 5.** Effect of dietary supplementation with fish oil (FO) or *Camelina sativa* seed oil (CSO) on lipoprotein concentration and size* (Mean values with their standard errors)

	Lipoprotein concentration and size												Student’s *t* test (*P*)		
	FO						CSO								ΔFO *v*. Δ CSO
	Start		End		Difference		Start		End		Difference		Start *v*. end		
	Mean	sem	Mean	sem	Mean	sem	Mean	sem	Mean	sem	Mean	sem	FO	CSO	
Concentration (nmol/l)															
Total VLDL + CM	40·9	2·7	39·0	2·9	−1·9	2·6	45·9	3·3	39·5	3·8	−6·3	4·0	0·5	0·1	0·2
Total LDL	965	60	946	57	−19	44	918	64	997	62	79	41	0·7	0·1	0·7
Total intermediate-density lipoprotein	151	14	147	16	−4	1·8	169	19	155	22	−14	25	0·8	0·6	0·1
Total HDL (×10^–3^)	32	31	31	1	−1	1	31·8	1	31·7	1	−0·1	0·9	0·2	0·9	0·4
VLDL + CM TAG	70·4	5·5	60·0	3·9	−10·4	4·2	68·3	1·8	60·2	5·1	−8·0	4·3	0·02	0·07	0·7
HDL-cholesterol	56·9	3·3	55·9	2·9	−0·9	2·2	56·7	2·8	58·4	2·7	1·6	1·8	0·7	0·4	0·4
Size (nm)															
Total VLDL + CM	51·0	1·4	49·8	1·0	−2·3	1·4	49·1	1·3	49·1	1·1	0·1	1·2	0·4	0·9	0·8
Total LDL	20·8	0·1	20·9	0·1	0·2	0·1	20·7	0·1	20·9	0·1	0·1	0·1	0·1	0·2	0·8
Total HDL	9·7	0·1	9·7	0·1	0·1	0·1	9·7	0·1	9·7	0·1	0·03	0·06	0·2	0·7	0·9

CM, chylomicrons.

* All data approximated a normal distribution. The change in mean particle concentration and size at the start and end of the trial and the effect of the type of oil on the mean change in lipoprotein concentrations from baseline were compared using Student’s paired *t* test.

### The effect of dietary supplementation with fish oil or *Camelina sativa* seed oil on EPA and DHA concentrations in erythrocyte lipids

Dietary supplementation with either FO or CSO significantly increased the proportion of EPA in erythrocytes by 27 and 40 %, respectively, which did not differ significantly between test oils (Table 6). There was no significant change in the proportion of DHA following supplementation with FO (Table 6). Supplementation with CSO induced a small (6 %) but statistically significant increase on the proportion of DHA in erythrocytes. The magnitude of the change in the proportion of DHA did not differ significantly between test oils.

**Table 6.** Effect of dietary supplementation with fish oil (FO) or *Camelina sativa* seed oil (CSO) on the proportions of EPA and DHA in erythrocyte total lipids* (Mean values with their standard errors)

	Proportion (% total fatty acids)							
	Start		End		Difference		Student’s *t* test (*P*)	
	Mean	sem	Mean	sem	Mean	sem	Change from baseline	Δ FO *v*. Δ CSO
EPA								
FO	1·1	0·1	0·3	0·1	0·3	0·1	<0·0001	0·3
CSO	1·0	0·1	0·4	0·1	0·4	0·1	<0·0001	
DHA								
FO	5·1	0·2	5·3	0·2	0·2	0·2	0·2	0·1
CSO	5·0	0·2	5·3	0·2	0·3	0·1	0·03	
Omega-3 index								
FO	6·2	0·2	6·7	0·2	0·5	0·2	0·02	0·2
CSO	6·0	0·3	6·8	0·2	0·7	0·2	<0·0001	

* All data approximated a normal distribution. The change in mean proportions of EPA and DHA, and omega-3 index between the start and end of the trial, and the effect of the type of oil on the mean change in proportions of EPA, DHA and the omega-3 index were compared using Student’s paired *t* test.

Supplementation with either FO or CSO induced a modest, statistically significant increase in the omega-3 index, 8 and 12 %, respectively (Table 6). There was no significant difference between test oils in the magnitude of the change in the omega-3 index.

## Discussion

The findings of this study show that consumption of EPA and DHA from CSO was as effective as FO in increasing the concentrations of EPA and DHA in plasma lipids and in erythrocytes.

The participants appeared to tolerate the test oils. The one participant who withdraw from the study did so because they found the FO unpalatable. This is consistent with unpalatability being a major factor in the reluctance of the UK population to consume oily fish^(8,9)^ and so supports the suggestion that consumption of CSO is a potential means to overcome unpalatability as a barrier to achieving recommended EPA + DHA intakes.

Consuming either CSO or FO increased the concentrations of EPA and DHA in the four plasma lipid classes that were measured. Thus, relatively short-term supplementation with the amount of EPA + DHA recommended by the UK government^(7)^ is effective in raising blood concentrations of these PUFA. In the fasting state, plasma PC and TAG reflect primarily hepatic synthesis and are carried by liver-derived lipoproteins, while the NEFA pool mainly reflects hydrolysis of TAG in adipose tissue^(24)^. Therefore, these findings suggest that consumption of CSO can induce comparable enrichment of EPA and DHA in hepatic and adipose tissue pools. The rank order of EPA incorporation in plasma lipids was PC ≡ CE > TAG > NEFA, while the rank order of the increment in DHA concentrations was PC > TAG ≡ NEFA irrespective of the test oil. Other studies have also reported differential incorporation of EPA and DHA into plasma lipid classes. For example, following dietary supplementation of men with FO, EPA was enriched in both plasma phospholipids and TAG, with a greater incorporation into phospholipids, but DHA was only enriched in TAG^(25)^. EPA has also been shown to be incorporated preferentially into phospholipids and CE, while DHA was incorporated predominately into phospholipid and TAG^(26)^. A study that investigated the incorporation of EPA and DHA consumed in different lipid structures into plasma lipids found that the increase in EPA and DHA was PC > TAG > NEFA in men and women^(15)^. It is unclear why different studies report differing distributions of EPA and DHA between plasma lipid classes, although this does not appear to be influenced by the dietary source or the relative amounts of EPA and DHA, the structure of the ingested lipid or the sex of the participants. However, such differences may have implications for understanding the metabolism of EPA and DHA and for the use of plasma lipids as biomarkers of EPA and DHA status.

There were no significant differences between test oils in the increments of EPA and DHA concentration in blood lipids or in erythrocytes after 8-week supplementation with 450 mg/d EPA + DHA provided as FO or CSO. This is in agreement with the pattern of postprandial incorporation of EPA and DHA into plasma lipids when consumed as FO or CSO^(14)^ and consistent with the view that CSO is as effective as FO in raising EPA and DHA concentrations when consumed in the amount recommended by the UK government. Thus, CSO is a potential alternative source of EPA and DHA for inclusion in the human diet. Moreover, CSO is potentially able to overcome the current barriers to achieving the level of EPA + DHA intake, in particular the perceived unpalatability of oily fish and dietary choices that exclude animal-derived foods.

The present findings indicate that consumption of CSO may confer the health benefits that have been attributed to FO, although the study was not designed specifically to test health-related outcomes because participation was restricted to healthy, normotriglycerideamic participants and was probably underpowered to detect health-related outcomes, and the level of EPA + DHA consumed was approximately 75 % lower than in studies which have reported health benefits including reduction in blood TAG concentration which typically requires intakes of >2 g EPA + DHA/d^(27)^. Nevertheless, the decrease in plasma TAG and VLDL + CM TAG concentrations induced by consuming either FO (−10·4 mmol/l) or CSO (−8·0 mmol), although the latter failed to reach statistical significance, was comparable to the reduction in plasma TAG induced in normotriglyceridaemic men who consumed purified 3·6 g/d EPA or DHA^(28)^.

The proportions of EPA and DHA in erythrocytes appear to reflect longer term intakes of these PUFA compared with more rapidly changing plasma lipids^(29,30)^, although the strength of this association may be modified by acyl exchange between erythrocyte and plasma lipids^(31)^, and by acyl remodelling and turnover which can be modified by dietary factors such as alcohol intake^(32)^. Nevertheless, the proportion of EPA + DHA in erythrocytes has been demonstrated to be a robust predictive biomarker of CVD risk^(5)^. The present findings show that there was no significant difference between test oils in the increase in the proportions of EPA and DHA in erythrocytes nor in the modest increase in the omega-3 index. Together, the changes in plasma and VLDL + CM TAG and in the omega-3 index suggest that consumption of CSO is potentially as effective as an equivalent intake of EPA + DHA provided as FO in ameliorating this risk factor and biomarker of CVD risk. Thus, it is reasonable to speculate that higher intakes of CSO over a longer period could induce clinically relevant changes in CVD risk.

The main limitation of the study is that the amount of EPA and DHA consumed per day was too low and the duration of the intervention was too short to test fully for beneficial effects of CSO in lowering plasma TAG concentration or to raise the omega-3 index. Moreover, there were too few participants to test for effects of age or adiposity on the incorporation of EPA and DHA into blood and cell lipids. Finally, there may be merit in a larger trial that includes different population subgroups such as patients with dyslipidaemia or inflammatory disease.

In conclusion, the present findings, together with those from a study of acute intake of CSO^(14)^, show that this oil from a transgenic plant is as effective as FO when consumed at an amount with equivalent EPA + DHA content in increasing blood and erythrocyte contents. Furthermore, the findings suggest that CSO may be able to confer health benefits that have been attributed to FO. Importantly, the transgenic oil was well tolerated. Thus, overall CSO is potentially an effective source of EPA and DHA for human consumption. Moreover, it does not incur the current challenges to the UK population achieving recommended intakes of these PUFA, namely concerns about palatability and contamination with environmental pollutants, and is consistent with dietary choices that exclude meat, while being potentially scalable without adversely affecting the marine environment.
